# Un traumatisme scrotal négligé révélant un rhabdomyosarcome embryonnaire para-testiculaire: à propos d’un cas

**DOI:** 10.11604/pamj.2018.30.107.15772

**Published:** 2018-06-11

**Authors:** Mustapha Ahsaini, Adil Mellouki, Khalid Ouattar, Hamid Azelmad, Soufiane Mellas, Jalaleddine Ammari, Mohammed Fadl Tazi, Mohammed Jamal Fassi, Moulay Hassan Farih

**Affiliations:** 1 Service d’Urologie, CHU Hassan II de Fès, Fès, Maroc

**Keywords:** Rhabdomyosarcome, traumatisme testiculaire, chimiothérapie, radiothérapie, chirurgie, Rhabdomyosarcoma, testicular trauma, chemotherapy, radiotherapy, surgery

## Abstract

Le rhabdomyosarcome paratesticulaire embryonnaire est une tumeur mésenchymateuse rare qui représente une urgence diagnostique et thérapeutique. Les formes localisées sont de pronostic favorable et un traitement multimodal fait appel à la chirurgie, à la polychimiothérapie et à la radiothérapie permettant d'aboutir à d'excellents taux de survie globale. Nous rapportons un cas colligé dans notre service de rhabdomyosarcome embryonnaire para testiculaire à cellules fusiforme, caractérisé par une confusion clinique, chez un jeune patient de 17ans, et à l'occasion du quel nous reprendrons l'ensemble des caractères spécifiques de cette pathologie.

## Abstract (en)

Embryonic paratesticular rhabdomyosarcoma is a rare mesenchymal tumor constituting a diagnostic and therapeutic emergency. Localized forms have a favorable prognosis. Multimodal treatment is the gold standard and it is based on surgery, multidrug chemotherapy and radiotherapy, with excellent overall survival rate. We here report the case of a 17-year old patient treated in our Department for embryonic fusiform cell paratesticular rhabdomyosarcoma causing clinical confusion. This study aims to highlight the specific features of this disease.

## Introduction

Les rhabdomyosarcomes para testiculaires (RMS-PT) sont des tumeurs mésenchymateuses rares, représentant environ 7% de tous les rhabdomyosarcomesde l'enfant et l'adulte jeune [1,2]. De part sa rareté, sa méconnaissance peut conduire à des situations tragiques potentiellement évitables. Le diagnostic différentiel se pose souvent avec les urgences scrotales. La prise en charge doit faire appel à une approche multimodale et multidisciplinaire. Aux meilleurs de nos connaissances c'est le premier cas clinique de Rhabdomyosarcome paratesticulaire révélé par un traumtisme scrotal négligé.

## Patient et observation

**Figure 1:**
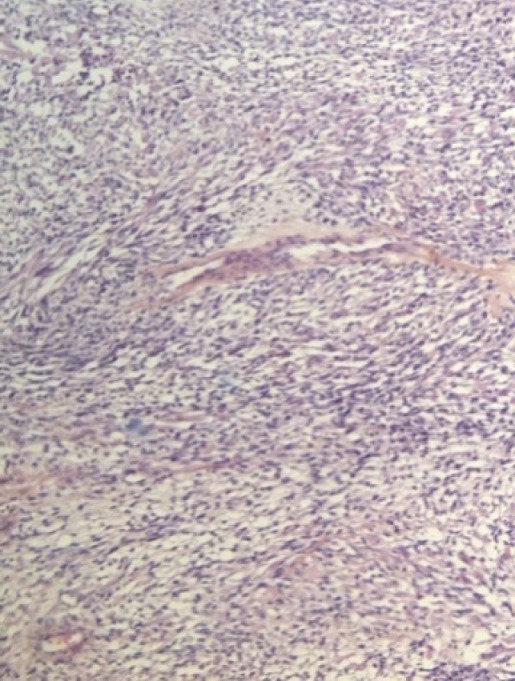
Image en microscopie à fort grossissement montrant des cellules fusiformes à fort pouvoir mitotique avec des rhabdomyoblastes

**Figure 2:**
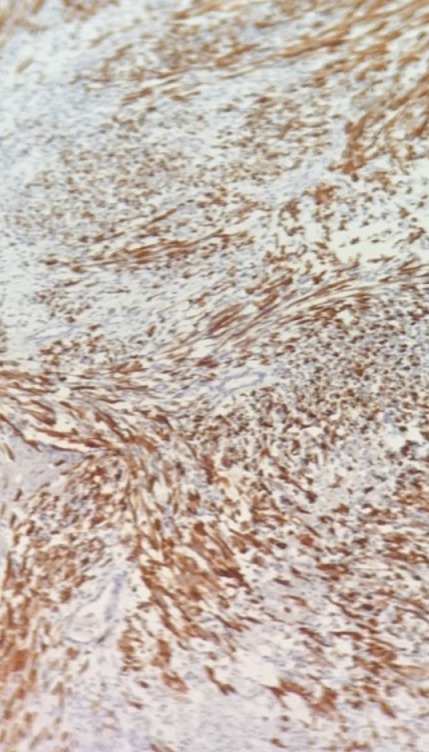
Immuno marquage montrant une positivité à l’anti-Désmine

**Figure 3:**
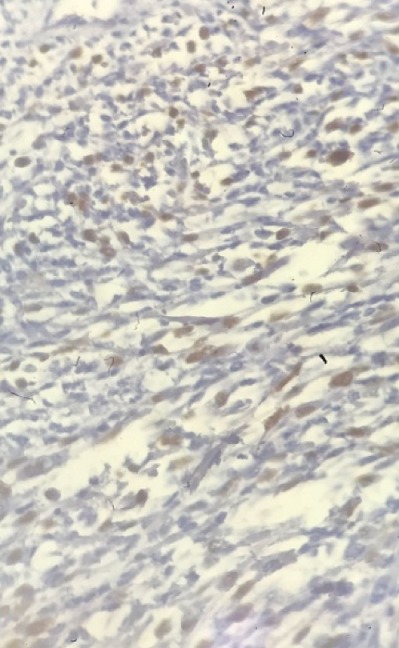
Immuno marquage montrant une positivité nucléaire à la myogénine

Un jeune homme âgé de 17 ans s'est présenté chez son médecin traitant avec une tuméfaction scrotale droite évoluant depuis 2 mois aux suites d'un traumatisme scrotale bénin négligé, l'absence de douleurs n'a pas motivé le patient a consulté que tardivement alarmé seulement par la progression en taille de la tuméfaction. L'échographie scrotale a objectivé une masse hétérogène solido-kystique intra scrotale refoulant le testicule droit qui est de taille normale et d'échostructure homogène sans individualisation de l'épididyme droit, le testicule gauche est normal. Le dosage des marqueurs tumoraux (BHCG, LDH, AFP) était normal. Le diagnostic d'une fonte purulente suite au traumatisme scrotal négligé a mené à la réalisation d'une orchidectomie droite par voie scrotale avec exérèse incomplète, dont l'examen anatomopathologique avec étude immun histochimique qui a conclu à un rhabdomyosarcome embryonnaire para-testiculaire à cellules fusiformes, envahissant le cordon spermatique dans la limite d'exérèse et la vaginale sans envahissement du testicule droit (Figure 1, Figure 2, Figure 3). La Tomodensitométrie Thoraco-abdomino-pelvienne (TDM TAP) réalisée un mois après la chirurgie a mis en évidence une masse scrotale de 7cm avec des adénopathies inguinales et iliaques externes de taille inférieure à 15mm, sans lésions secondaires à distance.

Du point de vue pronostique, nôtre patient diagnostiqué durant l'adolescence combine ainsi: 1) une tumeur vraisemblablement localisée en intra-scrotal sans signes de diffusion métastatique classée, T2N1M0, Grade 3 selon la classification de l'IRS (intergroupRhabdomysarcomastudy) vu le reliquat microscopique et la récidive locale; 2) une localisation paratésticulaire considérée comme de bon pronostic par rapport à d'autres localisations comme l'orbite ou la région paraméningée; 3) l'histologie de type embryonnaire considérée de meilleur pronostic par rapport au type alvéolaire ou encore pléomorphe.

**Figure 4:**
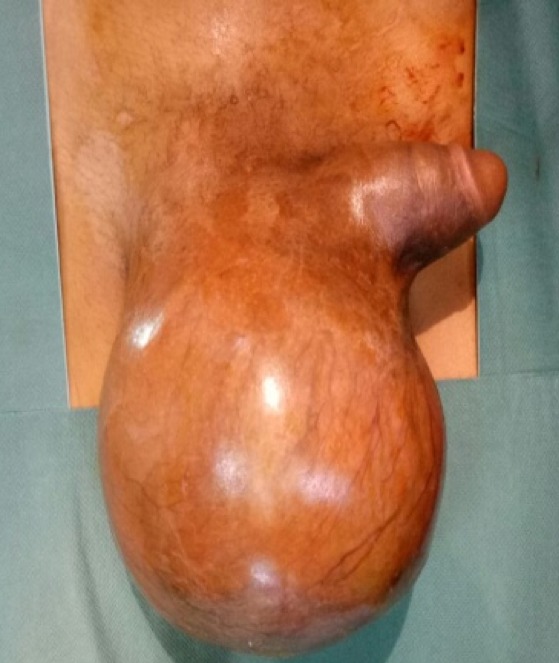
Récidive locale après chimiothérapie

**Figure 5:**
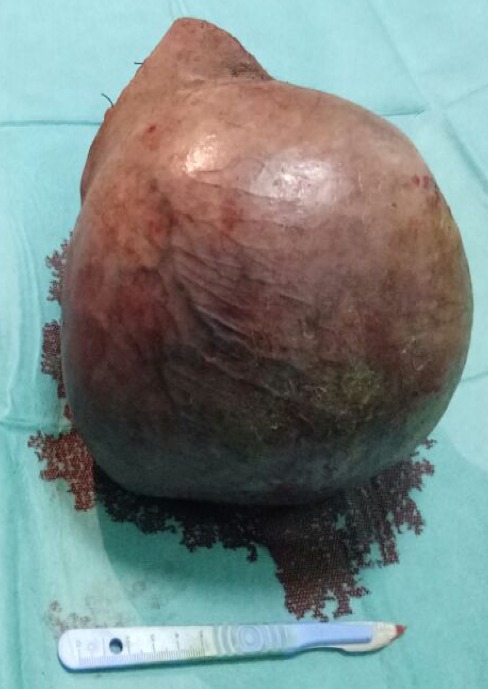
Pièce opératoire d’hémiscrotectomie

**Figure 6:**
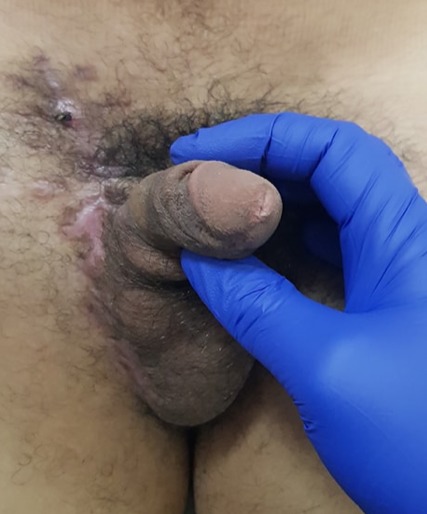
Etat local après hémiscrotectomie

Le patient a reçu 3 cures de chimiothérapie selon le protocole VAC (Vincristine Actinomycine et Cyclophosphamides) avec une scannographie d'évaluation à 2mois qui a objectivée une augmentation de la taille de la masse scrotale 9,5cm/7cm avec stabilité des ADP inguinales et iliaques externes, sans métastases (Figure 4). On a procédé à une reprise chirurgicale dans nôtre service, qui a consisté à une hémiscrotectomie droite jusqu'aux zones saines macroscopiquement associé a un curage inguinal et pelvien droitdont l'étude anatomopathologique n'a pas révélé d'envahissement scrotale ni ganglionnaire avec une réponse thérapeutique estimée à 70% (Figure 5). Un protocole de surveillance est établie sans récidive loco-régionale à ce jour avec un recul de 12 mois (Figure 6).

## Discussion

Le rhabdomyosarcome para testiculaire est une tumeur rare. Il représente 7% à 10% de l'ensemble des rhbabdomyosarcomes du tractus génito-urinaire, suivi par celui de la prostate et de la vessie. L'âge de survenue est caractérisé par deux pics d'incidence, le premier entre 2 et 5 ans, le second à l'adolescence [3]. La majorité de ces tumeurs sont de type embryonnaire (90%), le sous type alvéolaire ou pleiomorphe sont encore plus rares avec un pronostique plus sombre [2]. Il s'agit du 2eme cas traité dans notre institution durant 10 ans (4) en dehors des essais cliniques de l'IRS (intergroupe rhabdomyosarcoma study (I II II VI et V) et la SIOP (international society of pediatriconcology), la majorité des publications traitent des cas isolés [4]. La découverte clinique du rhabdomyosarcome paratesticulaire est souvent fortuite objectivant une masse intrascrotale indolore [5]. Devant une bourse aiguë non traumatique, le diagnostic est parfois difficile du fait de la présentation souvent confondue avec une torsion testiculaire, une tumeur testiculaire [5,6]. En relevant des situations particulières, cliniques ou thérapeutiques, ces publications soulignent la difficulté en pratique clinique d'évoquer un tel diagnostic. Ce constat est due non seulement à la rareté mais aussi au nombre de variables confondantes pouvant faire errer le diagnostic et retarder un traitement spécifique qui peut conduire à des situations à tout point de vue dramatiques. Comme le cas de nôtre patient où la notion de traumatisme bénin a rassuré faussement l'entourage et le premier médecin traitant, conduisant à un retard de diagnostique et le choix d'une voie d'abord scrotale initialement, formellement contre-indiqué en cas de suspicion de pathologie tumorale. Il faut signaler que dans quelques cas l'hydrocèle peut être une situation inaugurale [7]. L'échographie testiculaire est réalisée de première intention. Elle permet d'objectiver la masse intra scrotale, développée aux dépens des enveloppes testiculaires ou le cordon spermatique. Le testicule est le plus souvent intact [5,8].

Le dosage des marqueurs tumoraux (BHCG LDH AFP ACE) est souvent normal [9]. L'extension à distance se fait par voie lymphatique et hématogène. L'atteinte ganglionnaire lombo-aortique est rapportée dans 26% à 43% des cas. Le poumon, le foie et l'os sont les sites métastatiques les plus fréquents [5]. De ce fait La scanographie thoraco-abdomino-pelvienne contribue à l'appréciation de l'extension à distance de la maladie. La Scintigraphie osseuse, l'IRM ou la TEP-scanographie au FDG (fluorodésoxyglucose) ne font pas parti du bilan de routine et sont réservées à des présentations particulières [5,9]. La localisation paratésticulaire est considérée de bon pronostic par rapport à d'autres localisations comme l'orbite ou la région paraméningée [5,10]. Les RMS-PT embryonnaires ont un meilleur pronostic que les autres sous types notamment alvéolaires, botryoides, pleomorphe [4,5,10]. Le diagnostic anatomopathologique du rhabdomyosarcome paratesticulaire de type embryonnaire, repose sur confirmation de la nature musculaire de la tumeur et la présence inconstante de rhabdomyoblaste, l'immunomarquage permet d'affirmer le diagnostic par la positivité fréquente avec l'anti-desmine et les anticorps anti-myogénine [4].

La stadification des RMS paratesticulaires repose sur 3 critères qui sont [10]: 1) **le stade clinique:** déterminé à partir de données pré-thérapeutiques en fonction de la localisation primaire, la taille de la tumeur, la présence ou non de dissémination ganglionnaire ou de métastase à distance; 2) **Le Groupe:** déterminé par l'état local après une cure chirurgicale en fonction de l'évaluation histologique des marges de résection et des ganglions lymphatiques; 3) **La stratification du risque de récidive:** déterminé par une combinaison du stade clinique, le groupe et le type histologique.

Cette stadification a subi plusieurs adaptations reflétant l'évolution des essais cliniques de IRS (intergroupe rhabdomyosarcoma study), depuis IRS I En 1972 jusqu'au IRS V, qui visent à inclure des patients dans différents protocoles thérapeutiques en fonction du risque de récidive. Depuis sa création la survie globale à 5ans des patients avec une maladie localisée est passé de 55% avec l'IRS I à 90% avec l'IRS IV, ceci grâce à l'adoption d'un projet de soins multimodal combinant chirurgie extirpative, radiothérapie et /ou polychimiothérapie à base de Vancristine, dactinomycine et cyclophosphamide (VAC) [10]. Bien que l'histologie demeure le pilier du diagnostic et de classification, on a récemment mis l'accent sur des translocations génétiques, dont la plus élucidée est la fusion des gènes PAX7 (Ch1), PAX3 (Ch2) et le gène FOXO1 du chromosome 13 qui est observée dans le RMS de type alvéolaire et qui traduit un pronostic plus sévère.Ces anomalies génétiques seront incluses dans les prochains essais cliniques dans la perspective d'une classification pré-thérapeutique plus Précise [10,11]. L'orchidectomie par voie inguinale avec la résection du cordon spermatique est le traitement chirurgical standard des formes localisées [2], L'exérèse doit être large avec des marges de sécurité. Une approche par voie scrotale est jugée inadéquate en raison du risque de contamination de la peau par un résidu microscopique ou macroscopique et expose au risque de récidive locale [7]. Cette approche est néanmoins utilisée dans 25% des cas soit, dans le cas d'une confusion clinique avec des pathologies bénignes faisant préférer cette voie d'abord, comme le cas de notre patient, ou dans le cadre d'une approche d'excision primaire suivie ou non d'une hemiscrotéctomie en fonction de l'apparition ou non d'une récidive locale, cette approche a été abandonnée vu la supériorité du protocole multimodal en matière de contrôle de la maladie initiale [10,12-14]. La polychimiothérapie permet d'éradiquer les métastases occultes [7]. Elle est indiquée pour tous les groupes pronostiques avec une amélioration significative de la probabilité de survie globale et de la survie sans progression. Plusieurs protocoles de chimiothérapie ont été utilisés dans le traitement des rhabdomyosarcomes. Les associations IVE (ifosfamide, vincristine, étoposide) ou IVA (ifosfamide, vincristine, dactinomycin) ou VAC (vincristine, dactinomycin, cyclophosphamide) sont les plus utilisées. L'essai de l'IRS IV a montré que le protocole VAC était aussi efficace que les protocoles IVA et IVE [8,10]. La radiothérapie est une arme thérapeutique importante dans la prise en charge des rhabdomyosarcomes, elle permet d'améliorer le taux de contrôle local et par conséquent le pronostic. Le taux de contrôle local des rhabdomyosarcomes irradiés de groupe II est supérieur à 90% [8,10].

Les séquelles tardives de la radiothérapie représentent un facteur limitant pour les enfants. Cependant, les avancées technologiques ont permis d'améliorer les techniques d'irradiation et limiter les effets secondaires (IMRtintensitymodulated Radiation therapyou VMAT volume modulated arc therapy) en épargnant les organes à risque adjacents [15]. De part sa survenue dans une population jeune (enfants, adolescents et jeunes adultes), il faut souligner l'importance de la préservation de la fertilité qui peut être affectée par la toxicité de certains agents de chimiothérapie notamment les cyclophosphamides qui altèrent l'épithélium germinal et leur utilisation est corrélée à une fréquence non négligeable d'hypogonadisme et d'anomalies de spermogramme chez la population traitée (58% de cas d'azoospermie et 28% de cas d'oligospermie). Un effet secondaire qui est dose dépendant [16,17]. Une question pertinente qui doit préoccuper tout intervenant dans le parcours de soins, surtout dans notre pays où la place de la préservation de la fertilité du patient oncologique occupe une place très restreinte devant la rareté des centres d'auto-préservation; CECOS: les Centres d´étude et de conservation des œufs et du sperme humains.

## Conclusion

Le RMS-PT est une tumeur mésenchymateuse rare qui représente une urgence diagnostique et thérapeutique. Les formes localisées sont de pronostic favorable et un traitement multimodal permet d'aboutir à d'excellents taux de survie globale. Plusieurs voies thérapeutiques sont en voie d'exploration, notamment les thérapies ciblées compte tenu du profil moléculaire particulier des rhabdomyosarcomes du tractus uro-génital. Avec l'avènement des tests génomiques et l'amélioration des modalités d'imagerie et de traitement, L´enjeu majeur des années à venir sera très certainement de pouvoir adapter les traitements aux anomalies moléculaires présentes chez un individu donné au moment de la décision thérapeutique. Dans la perpective d'une médecine de précision et un meilleur contrôle de la maladie avec moins d'effets secondaires.

## Conflits d’intérêts

Les auteurs ne déclarent aucun conflit d'intérêts.
